# Object speed perception during lateral visual self-motion

**DOI:** 10.3758/s13414-021-02372-4

**Published:** 2021-10-26

**Authors:** Björn Jörges, Laurence R. Harris

**Affiliations:** Center for Vision Research, York University, 4700 Keele Street, Toronto, ON M3J 1P3 Canada

## Abstract

Judging object speed during observer self-motion requires disambiguating retinal stimulation from two sources: self-motion and object motion. According to the Flow Parsing hypothesis, observers estimate their own motion, then subtract the retinal corresponding motion from the total retinal stimulation and interpret the remaining stimulation as pertaining to object motion. Subtracting noisier self-motion information from retinal input should lead to a decrease in precision. Furthermore, when self-motion is only simulated visually, self-motion is likely to be underestimated, yielding an overestimation of target speed when target and observer move in opposite directions and an underestimation when they move in the same direction. We tested this hypothesis with a two-alternative forced-choice task in which participants judged which of two motions, presented in an immersive 3D environment, was faster. One motion interval contained a ball cloud whose speed was selected dynamically according to a PEST staircase, while the other contained one big target travelling laterally at a fixed speed. While viewing the big target, participants were either static or experienced visually simulated lateral self-motion in the same or opposite direction of the target. Participants were not significantly biased in either motion profile, and precision was only significantly lower when participants moved visually in the direction opposite to the target. We conclude that, when immersed in an ecologically valid 3D environment with rich self-motion cues, participants perceive an object’s speed accurately at a small precision cost, even when self-motion is simulated only visually.

## Introduction

When observing a moving target while moving, the same retinal speeds can correspond to vastly different physical velocities. When an observer moves in the same direction, parallel to a moving object, the retinal speed of the object is partially cancelled out, and when they move in the direction opposite to the object, the retinal stimulation due to self-motion may be added to the retinal speed of the object. To obtain an accurate estimate of the object’s velocity, observers must therefore obtain an accurate estimate of their own velocity and subtract or add the consequences of this movement to the retinal motion of the target. More specifically, the Flow Parsing Hypothesis (Dupin & Wexler, 2013; Rushton & Warren, 2005; Warren & Rushton, 2008, 2009) posits that, to estimate object motion from ambiguous retinal input representing the sum of object and self-motion, observers first compute which components of retinal stimulation are caused by their own motion in the environment. Then, they subtract this self-motion information from the overall retinal stimulation and attribute the remaining stimulation to object motion in the scene. When self-motion is experienced only visually while undergoing no physical motion, the visual motion creates a conflict between visual and vestibular inputs as a result of which self-motion is likely to be underestimated, leading to biases in judgments of object velocities, although oddly this has never been quantified for horizontal translation. The effect has been shown to some extent for vertical observer and object translation (Dyde & Harris, 2008), as well as for rotating observers (Garzorz et al., 2018; Hogendoorn et al., 2017; Probst et al., 1995) and motion-in-depth(Gray et al., 2004). Furthermore, it has been argued that self-motion information is noisier than retinal information concerning object motion, especially when observers have only visual information about their own movement at their disposal (Fetsch et al., 2010). Subtracting noisy self-motion information from retinal motion in order to obtain an estimate of target velocity should thus decrease precision (Dokka et al., 2015). Such a subtraction process is relatively straightforward for the consequences of angular self-motion, but for lateral motion, the geometry requires additional computations involving estimates of the distance of the object to the observer and the direction of object motion relative to the observer’s motion. More specifically, observers need to first estimate their own motion in an allocentric world frame by using retinal stimulation attributable to the induced motion of static objects in the environment and other sensory and efferent information such as vestibular activity. Then this estimate needs to be used to generate an estimate of the retinal stimulation expected to be caused by the observer’s motion. This estimated retinal stimulation due to self-motion is then subtracted from the total retinal stimulation, which allows the remaining retinal stimulation to be interpreted as external object motion. The process is known as “flow parsing,” in which the different aspects of the total optic flow are attributed to these different causes.

It is important to note that flow parsing is only necessary when humans need to represent the kinetic properties of their environment in an allocentric frame. For computations performed in an egocentric frame, it is generally sufficient to time interceptive actions and avoid collisions according to the velocity of the target relative to the observer. It is true that ecological, optic-flow-based heuristics have successfully explained humans’ performance in paradigmatic cases such as the outfielder problem (Fink et al., 2009; Wilson & Golonka, 2013). However, humans are able to recover, represent, and use the physical parameters of their environment in a variety of tasks (Burr et al., 2007; Fajen et al., 2013; Ilg et al., 2004; Wexler, 2003).

There are two major sources of information about passive self-motion: visual and vestibular cues (Fetsch et al., 2010), which are integrated according to their relative reliability (Fetsch et al., 2009). How much each sense contributes to the global percept of self-motion seems to depend on different parameters, such as the task and the self-motion profile. Dokka et al. (2015), for example, found for direction judgments of a probe presented in the fronto-parallel plane during lateral observer motion that vestibular information in the absence of visual information led to a vast underestimation of self-motion. Visual information only elicited a higher accuracy, and having both visual and vestibular cues available increased accuracy only marginally beyond accuracy for visual information only. In a more direct test of perceived self-motion, Harris et al. (2000) found that vestibular stimulation evoked by moving the observer through the environment was an extremely potent cue to self-motion, which induced a vast overestimation of the distance moved. Visual cues to self-motion were efficient, too, but less so than vestibular cues. However, depending on stimulus parameters such as simulated acceleration, visual cues alone can also lead participants to overestimate their movement (Redlick et al., 2001). For active self-motion (i.e., movements initiated by the observer such as walking through the environment), efference copies and proprioceptive information can serve as further cues. For example, judgments about the distance travelled seem to be more reliable if motion was self-generated as opposed to experienced passively (Becker et al., 2002; Frissen et al., 2011; Jürgens & Becker, 2006).

Remarkably, the literature is quite sparse with regards to assessing object motion during lateral, visually simulated observer motion: Warren and Rushton (2007) found that translational visually evoked self-motion led observers to perceive the trajectory of a linearly moving probe as tilted towards the direction of the simulated translation. MacNeilage et al. (2012) showed that vestibular cues could help distinguish self-motion from object motion, especially for lateral observer motion. Similarly, Dokka et al. (2015) investigated the extent to which observer motion (visual cues only, vestibular cues only, and both visual and vestibular cues) influenced the judged direction of vertical downwards motion with a small lateral component. They found biases in line with insufficient compensation for self-motion in all observer-motion conditions, as well as decreases in sensitivity. Niehorster and Li (2017) quantified the extent to which flow parsing was complete for straight-ahead self-motion by having participants judge the direction of a probe that moved vertically upwards. Importantly, all these studies used direction judgments as proxies to probe the completeness of flow parsing, while a direct psychophysical investigation of perceived *velocities* is notably missing from the literature. Furthermore, while some of these studies presented their stimuli in compelling stereo 3D, none immersed the observer in a virtual environment. It is not unlikely that flow parsing is facilitated by a more realistic environment. This study aimed to shed light on the extent to which visually evoked self-motion influences perceived lateral object *speed* in a naturalistic setting. This is particularly relevant as the visual system has been shown to use velocity information to extrapolate object trajectories to compensate for noisy online information and neural delays (Aguado & López-Moliner, 2019; Aguilar-Lleyda et al., 2018; Jörges & López-Moliner, 2019; López-Moliner et al., 2010). The aim of this project is thus to verify the impact of visually simulated observer motion on accuracy and precision for object speed judgments during lateral translation, which will further our understanding of flow parsing and help us understand the conditions under which flow parsing is incomplete. More specifically, our hypotheses are:

- When the observer experiences *no visual self-motion* during object motion observation, we expect the *highest accuracy* of speed estimation.
- When visual observer motion is simulated *opposite to the object motion (e.g., observer moves to the right, object moves to the left)* during object motion observation, we expect them to *overestimate the observed speed.*
- When visual observer motion is *simulated in the same direction as the target (e.g., both observer and target move to the right)* during object motion observation, we expect them to *underestimate the observed speed*.
- We expect the *precision to be lower* when the participant experiences visually simulated observer *motion during object motion observation* relative to when they are static.

## Methods

### Participants

We recruited 30 participants (see power analysis) through word-of-mouth, posts in online VR communities, and the company XpertVR. Participants we recruited directly (n = 17) received a compensation of CAD$30 (or equivalent in the local currency at the time of payment) as an amazon.com gift card. Participants (n = 13) recruited through XpertVR received a compensation of US$50. Due to the culturally independent nature of the phenomenon under study, we do not believe our results are likely to be relevantly skewed by WEIRD people effects (Henrich et al., 2010). To test for stereo-blindness, participants performed a short custom experiment in VR in which they had to tell which of two rectangles ahead of them appeared closer. The rectangles were matched in optical size such that only stereo cues could provide information about their relative distances. The difference between the two rectangles was simulated to be 200 arcseconds and participants had to guess 16 out of 20 trials correctly. This experiment, along with some additional explanations about the geometry of the scene, is available for download here (on GitHub: https://github.com/b-jorges/Stereotest). The project has received ethics approval from the Human Participant Ethics Review Sub-Committee at York University. Informed consent was obtained from all participants and the experiment was conducted in accordance with the Code of Ethics of the World Medical Association (Declaration of Helsinki).

### Apparatus

All the experiments were performed in virtual reality with participants remaining physically static and seated. We programmed the stimuli in Unity (2019.2.11f1), while object motion, visually simulated observer motion, and the psychophysical staircases were controlled in C# via its integration with Unity. The Unity project is available on the Open Science Framework (https://osf.io/m6ukw/). Stimuli were presented in a VR (virtual reality) headset. Participants used the equipment available to them at home, among which were Oculus Rift CV1, Oculus Rift S, Valve Index, Oculus Quest, Oculus Quest 2, and HTC Vive. Participants responded using the keyboard on their computers.

### Administration of the experiment

Due to the global COVID-19 pandemic of 2020–2021, data collection could not occur in a lab setting as planned. Rather, we opted for an online approach: we recruited participants who had suitable VR equipment at home and were willing to complete our experiment. We recruited participants through word-of-mouth, posts on social media such as Twitter and Reddit, and the company XpertVR. To guide our online participants through the experiment, we provided instructions both as a PDF document (see GitHub: https://github.com/b-jorges/Motion-Perception-during-Self-Motion/blob/master/Instructions/Instructions%20Seeing%20while%20moving.pdf) and as a video (uploaded on YouTube: https://youtu.be/jN141KbNOWA). After they emailed back their signed informed consent forms, they were sent a link to download a zip package with the four parts of this experiment (a stereo-blindness test, a training for the main experiment, the main experiment, and an assessment of how they perceived visually simulated self-motion in our stimulus), which they were asked to run on their own hardware. Participants first had to complete the stereo-blindness test and the training for the main experiment, which also served as a screener to verify whether they were performing the task correctly. All participants who sent back their data and who were thus included in our analyses completed both checks successfully. After these two pretests, they performed the main experiment followed by an assessment of their perception of self-motion. They then sent the data back to us and were paid for their time with a CAD$30 (or the local equivalent) Amazon gift card. Participants recruited through XpertVR received US$50 (or the local equivalent).

### Setup

Our experiment consisted of a Two-Interval Forced-Choice Task where participants were asked to indicate which of two intervals contained objects moving at the higher speed.

#### Environment and general layout

**Fig. 1:**
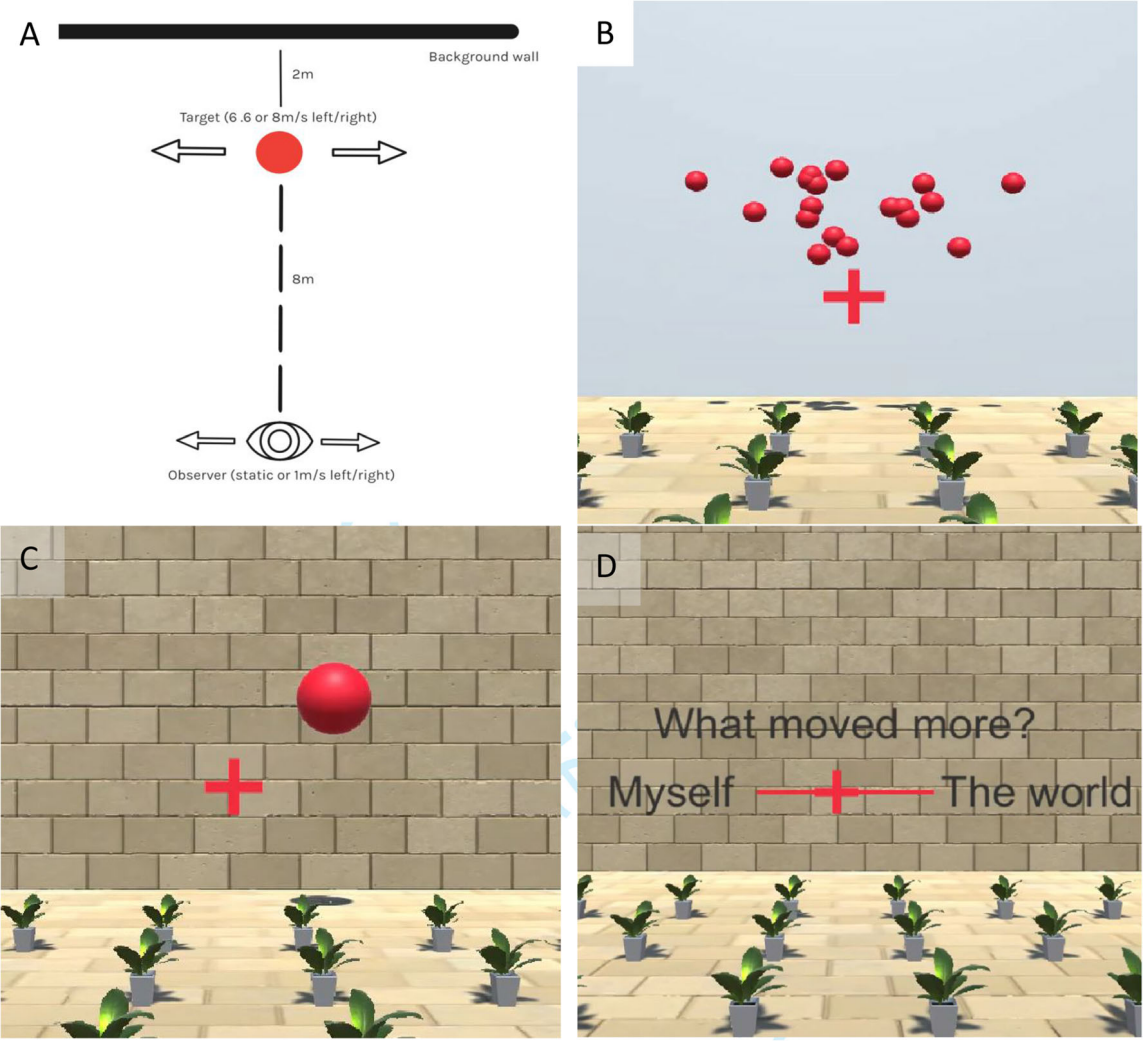
**a** Top view of the stimulus scene in one of the test trials. The red circle represents the target, which starts on one side of the midline (see text for details) and moves laterally at 6.6 or 8 m/s for 0.5 s, that is, 3.3 or 4 m. The stylized eye indicates the position of the observer, who can be static or move to the left or to the right for 0.5 s with a Gaussian motion profile and a mean speed of 1 m/s. The target is 8 m away from the observer and 2 m in front of the background wall. **b** Screenshot from the program during presentation of the dot cloud in the untextured wall condition (“Blank Backdrop”). **c** Screenshot from the program during presentation of the big target in the textured wall condition (“Textured Moving Backdrop”). **d** Screenshot from the self-motion judgment conducted after the main body of the experiment. A short sequence of the main experiment can be downloaded from GitHub at: https://github.com/b-jorges/Motion-Perception-during-Self-Motion/blob/master/Figures/Main%20experiment.mp4. A sequence from the self-motion judgment part of the experiment is available from GitHub at: https://github.com/b-jorges/Motion-Perception-during-Self-Motion/blob/master/Figures/Selfmotion%20judgement.mp4

Participants were immersed in a virtual 3D environment that included depth cues from lighting, shadows, and the scale of the textures of the floor and the wall backdrop. The ball appeared to the left of the observer if it moved to the right, and to the right of the observer when it moved to the left. The exact position was determined by target speed and visual observer motion (see Eq. 1). See Fig. 1a for a diagram of the visual scene and Fig. 1c for a screenshot from the experiment; a short sequence of the experiment can be downloaded from GitHub at: https://github.com/b-jorges/Motion-Perception-during-Self-Motion/blob/master/Figures/Main%20experiment.mp4.

#### Targets and visually simulated observer motion

In one interval participants were presented with a ball with a diameter of 0.33 m at a simulated distance of 8 m in front of them, travelling with 6.6 or 8.0 m/s (two target-motion profiles). Participants were seated and the camera was simulated 1.3 m above the ground. The targets were simulated 2 m above the ground. The direction in which the targets moved was chosen randomly on each trial. During this interval, participants were either static (“Static with Textured Backdrop”) or experienced visually simulated observer motion to the left or to the right with a Gaussian speed profile (three self-motion profiles), accelerating until reaching peak speed after 0.25 s and then slowing down until coming to a halt at 0.5 s (“Simulated Observer Motion with Textured Backdrop”). The position in time *x(t)* was given by a cumulative Gaussian distribution with a mean of 0.25 s and a standard deviation of 0.08 s divided by 2, multiplied by -1 for trials with visually simulated observer motion to the left. That is, participants were moved visually 0.5 m over the course of 0.5 s, which amounts to a mean speed of 1 m/s. The target’s initial position was shifted away from the observer for motion in the same direction, and towards the observer when the observer’s visual motion occurred opposite to the target motion, such that observer and target motion were symmetrical, i.e., the distance between observer and target at the beginning was the same as the distance at the end of the trial. To achieve this, the starting position of the target was computed in the following manner:

1 $$ {x}_{initial}=0.5\ast \left( Distanc{e}_{observer}-{v}_x\ast t\right) $$

where *x*_*initial*_ denotes the initial position of the target relative to the observer, *Distance*_*observer*_ is the lateral distance in which the observer is simulated to move, *v*_*x*_ is the horizontal speed of the target, and t is the duration of the motion interval (0.5 s).

#### Staircase

In the other interval, participants were shown a cloud of smaller moving balls each with a diameter of 0.1 m as comparison. The balls appeared 1.25 m to the left of the observer (if the big target in the same trial moved to right) or to the right of the observer (if it moved to the left), then moved in the same direction as the big target and disappeared after having travelled 2.5 m. They were spread out vertically over a distance of 1 m. Ten to 15 balls were visible at any given moment. Observers were asked to maintain their gaze on a fixation cross that was continuously displayed straight ahead of them (i.e., also during visually evoked self-motion), 0.8 m under the target (see Fig. 1b). The speed of these smaller balls was controlled by a PEST staircase and constant across their lifetime. We employed two staircases for each combination of visually stimulated self-motion (left, right, or static) and object motion (6.6 and 8 m/s), one of which started 33% above the target’s speed, and the other one 33% below target speed (two staircases for each combination of target motion and self-motion). The direction (left to right or right to left) was chosen randomly for each trial. Thus, there was a total of two target speeds x three motion conditions x 2 = 12 staircases. The step sizes were governed by the following rules (Taylor & Creelman, 1967): the initial step size was 1.2 m/s. For the first five trials for each PEST, the step size was maintained. Starting from the 11th trial, after a reversal (participants answered “PEST is slower” in the second-to-last trial and “PEST is faster” in the last trial or vice-versa), the step size was halved. After the second same answer, the step size was maintained. After the third same answer, the step size was either maintained when the step size had been doubled before the last reversal or doubled when the step size had not been doubled before the last reversal. After four same answers, the step size was always doubled. Each PEST ended when it converged (five consecutive trials with step sizes lower than 0.1) AND participants had judged at least 20 trials of the staircase. If the staircase did not converge, the PEST was terminated after 27 trials. The experiment ended when all PESTs, including the control PESTs (see *A possible confound: Induced motion* section below), had terminated. This took about an hour overall including instructions and breaks, which participants could take every 15 min.

Before starting the actual data collection, participants perform a training session with one PEST where the big target moved at 4 m/s. Participants were asked to repeat the training if the step size in any of the last five trials was above 0.3 m/s. If they still failed to meet the criterion after a second repetition they were excluded from the experiment. No participant reported failing this task.

#### Intended interpretation of visually simulated observer motion and task

Our experiment critically depended on our participants perceiving themselves as moving rather than the world as moving. We were furthermore interested in participants making the velocity judgments relative to the world, not relative to themselves, and we assume that our instruction will make sure of this. However, there is a possibility that either of these assumptions will not hold. This gives rise to four different scenarios during visually simulated observer motion:

1. Participants perceive the world as static and themselves as moving and judge object speed relative to the world. This is the intended case.
2. Participants perceive themselves as static and the world as moving and judge object speed relative to the world. In this case we would find no effect of visually simulated observer motion at all, that is, there would be no differences between visually simulated observer motion and a (visually) static observer.
3. Participants perceive the world as static and themselves as moving and judge object speed relative to themselves. In this case, participants would add the speed of visually simulated observer motion fully onto the target speed, that is, the PSE would be shifted by roughly the mean value of the visually simulated observer motion.
4. Participants perceive themselves as static and the world as moving and judge object speed relative to themselves. In this case, participants would also add the speed of visually simulated observer motion fully onto the target speed, that is, the PSE would be shifted by roughly the mean value of the visually simulated observer motion.

To rule out scenarios (2) and (4), we had our participants judge to what extent they felt themselves or the world moving. They were only included into the confirmatory analyses if they had a mean rating between -1 and -0.6 in their judgments about perceived self-motion versus world-motion (see below), indicating that they fully or mostly perceived themselves to move, rather than the world. To achieve that, participants made their judgments relative to the world and not to themselves, thus ruling out scenario (3), we gave them very clear instructions to this effect.

It is also possible that participants judge motion partially relative to the world and partially relative to themselves. This seems to be roughly equivalent to a scenario where participants judge motion relative to the world but fail to compensate fully for self-motion in their object speed judgments. Shifting from an observer-centered reference frame to a world-centered reference has been suggested as the mechanism behind the accuracy-precision trade-off observed by Dokka et al. (2015): in this view, transposing a percept into a world-centered reference frame increases accuracy, that is, enhances compensation for self-motion, at the cost of a decrease in precision.

#### A possible confound: Induced motion

Induced motion occurs when a stimulus is contained within a moving reference frame. Even in a VR 3D presentation, a texture background might be construed as such a reference frame and therefore induce motion. Induced motion would bias perceived speed in the opposite direction to the background motion and could therefore mask potential effects of self-motion. That is, any induced motion would tend to cancel out incomplete compensation for self-motion that would otherwise reveal itself as motion in the same direction as the background, leading to an overall accurate speed estimate. The strength of induced motion depends on several factors: The adjacency principle, which states that stimuli that are closer together in space (in all three dimensions) lead to stronger induced motion (Gogel & Koslow, 1972; Gogel & MacCracken, 1979). Furthermore, enclosure seems to be important. Experiments on induced motion without a full rectangular reference frame are rare, but even moving dots have been shown to induce some motion, albeit to a much lesser extent than a full frame. Brosgole and Whalen (1967), for example, found that induced motion was halved when using a dot as the inducing stimulus moving 0.2° from the induced stimulus, in comparison to the effect of a full rectangular frame at the same distance. Duncker (1929) observed that a horizontal line moving horizontally induced much less motion than a vertical line moving horizontally or a full rectangle, which is arguably a scenario that comes closest to our display.

##### Control conditions: Setup

To account for this possible confound, we added two additional conditions. In the first condition (“Blank Backdrop”), we minimized possible induced motion effects by using an untextured wall backdrop (Fig. 1b). Motion might still be induced by the other objects in the visual scene, but in the absence of any traditional frame, induced motion should be minimal. All other experimental parameters were the same as for the main experiment. In the second condition (“Textured Moving Backdrop”), we aimed to minimize perceived self-motion while keeping the induced motion component of the effect intact. We achieved this by moving only the (textured) wall backdrop of the stimuli, while keeping the rest of the visual scene (textured floor, context objects) static. The wall backdrop moved with the same motion profile as the observer was moved visually in the other conditions. This added another 14 staircases (six for the *Textured Moving Backdrop* condition, and eight for the *Blank Backdrop* condition because, for the latter, we also added two PESTs without visually simulated observer motion) that were interleaved in a random order with the staircases described above.

##### Probing perceived self-motion

To assess whether this manipulation worked as intended, after conclusion of the main body of the experiment, we showed the participants the different conditions (*Simulated Observer Motion with Textured Backdrop*, *Blank Backdrop*, and *Textured Moving Backdrop*) without the object motion. After they experienced the stimulus (i.e., visually simulated observer motion in the full room, visually simulated observer motion with the untextured wall background, or no visually simulated observer motion, but a moving wall backdrop), we asked them to rate on a continuous scale within the virtual environment (see Fig. 1d) to which extent they had perceived themselves or the world/wall as moving. Figure 1d shows a screenshot taken from the judgment phase of the task. We repeated this procedure four times for each condition and direction for a total of 24 trials. This took about 2 min. We included only those participants whose mean ratings per condition were within 0.4 of the expected value (i.e., between -1 and -0.6, indicating a high degree of observer motion, for *Blank Backdrop*; between 0.6 and 1, indicating a high degree of wall motion, for *Textured Moving Backdrop*; and between -1 and -0.6, indicating a high degree of observer motion, for the main test condition, *Simulated Observer Motion with Textured Backdrop*).

### Data analysis

#### Outlier analysis and exclusion criteria

We first filtered out trials where the pest speed was more than 1.5 times higher than the standard speed, which amounted to trials where participants accidentally pressed the wrong button early on the in the staircase (1% of all staircases). To filter out staircases that did not converge satisfactorily, we computed the average test speed of the last ten trials of both threads pertaining to each staircase. When the average velocity of both threads was more than 3 m/s apart, we excluded the staircases. This accounted for 4.4% of the remaining staircases. *Please note that this outlier analysis was added post hoc* because we failed to include an outlier analysis in the preregistration. For full transparency, we also included our main analyses over the unfiltered dataset in Appendix B. Please find the script we used for this preprocessing step on GitHub at: https://github.com/b-jorges/Motion-Perception-during-Self-Motion/blob/master/Final%20Paper%20Data%20Preprocessing.R.

**Fig. 2:**
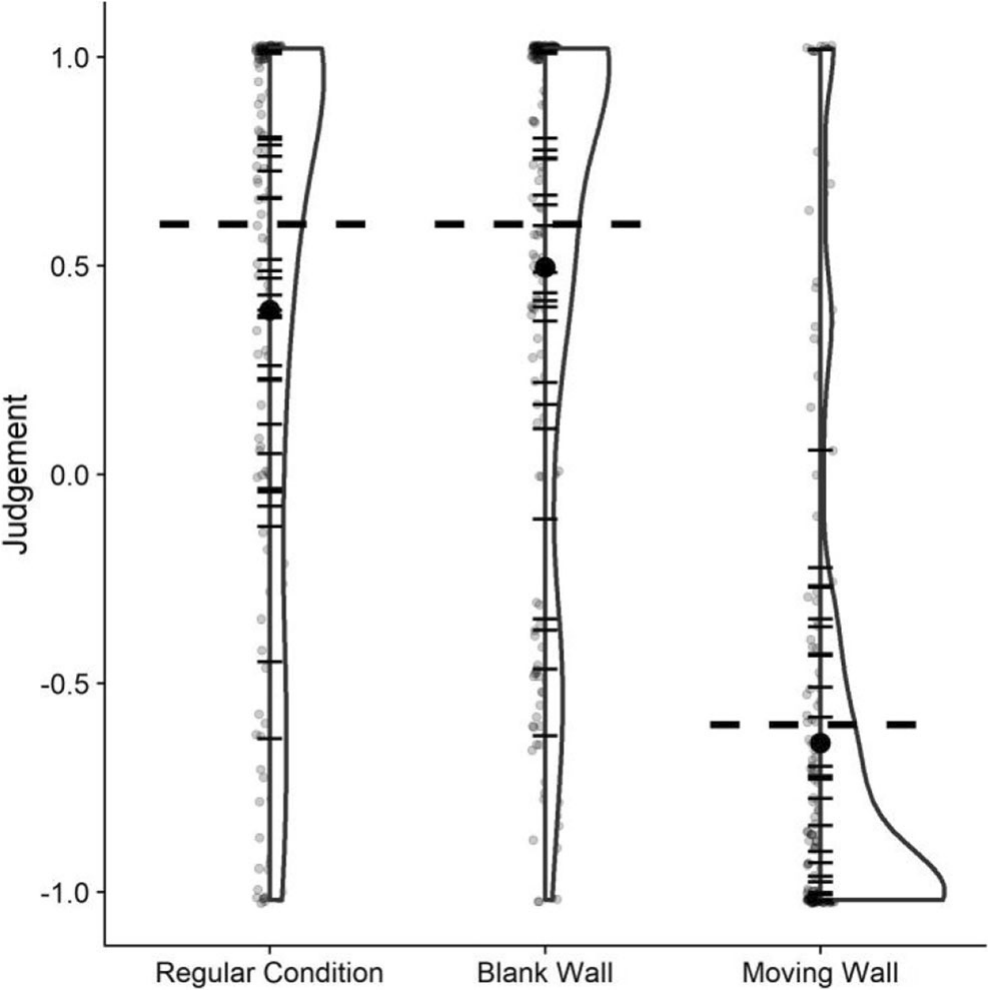
Distributions of judgments for the self-motion judgment task. For the Regular Condition and the Blank Wall condition, a rating of above zero meant that the participant perceived themselves to move rather than their environment, while a rating of below zero meant that they perceived the environment to move rather than themselves. For the Moving Wall condition, a rating above 0 means that they perceived themselves to move rather than just the wall backdrop, while a rating below zero means that they perceived the wall to move rather than themselves. The horizontal bars represent the mean judgment for each participant; the bolt black dot shows the mean across all participants; the translucent grey dots represent one data point each. Finally, the horizontal dashed lines correspond to the cut-off criterion we had established in the preregistration. For the Regular Condition and the Blank Wall condition, we expected participants to make judgments above 0.6, while we expected judgments of below -0.6 for the Moving Wall condition

Further, we had planned to only include those participants who satisfied our criterion in the self-motion perception task: participants needed to give the regular condition and the blank wall condition an average rating of > 0.6, indicating that they mostly perceived themselves to move rather than the world; and a rating of < -0.6 was required for the moving wall condition, indicating that they perceived the wall to move rather than themselves. See Fig. 2 for the distribution of average responses. Descriptively, only about half of the participants satisfied our criterion for each condition. Since data collection for this study occurred during the COVID-19 pandemic of 2020–2021, which made access to participants for a study in VR considerably harder, we decided to conduct an exploratory analysis as to whether there were significant differences between the participants that satisfied out criterion and those that did not.

For this exploratory analysis, we used generalized linear mixed modelling (GLMM), implemented in the lme4 package (Bates et al., 2015) for R, according to the recommendations in (Moscatelli et al., 2012). To accommodate the non-linear character of psychophysical data, we used a probit link function. We first fitted a test model with “Subject Motion” (a ternary variable with the levels “Static,” “Same direction,” and “Opposite directions”), “*Speed*_*Ball Cloud*_” (the speed of the ball cloud) and “Satisfied Criterion” (a binary variable with the values “Satisfied Criterion” and “Did not satisfy Criterion”) as well as all interactions as fixed effects, as well as random intercepts and random slopes for “*Speed*_*Ball Cloud*_” and “Subject Motion” per speed of the comparison stimulus (“*Speed*_*Big Target*_) and per Participant (“*Subject*“) as random effects. We fitted this model across the whole dataset, neglecting the differences between the Conditions (Main Condition, Blank Wall condition, and Moving Wall condition) to keep the computational complexity within reasonable limits. We used a probit link function. In lme4 syntax, this model reads as follows:

2 $$ Response\sim Spee{d}_{Ball\ Cloud}\ast Subject\ Motion\ast Met\  Criterion+\left( Spee{d}_{Ball\ Cloud}+ Subject\ Motion\right|\kern0.5em Subject\left)+\left( Spee{d}_{Ball\ Cloud}+ Subject\ Motion\right| Spee{d}_{Big\  Target}\right) $$

We additionally fitted a null model with the same specifications, but without “Satisfied Criterion” and the respective interactions as fixed effects. This null model reads as:

3 $$ Response\sim Spee{d}_{Ball\ Cloud}\ast Subject\ Motion+\left( Spee{d}_{Ball\ Cloud}+ Subject\ Motion\right|\kern0.5em Subject\left)+\left( Spee{d}_{Ball\ Cloud}+ Subject\ Motion\right| Spee{d}_{Big\  Target}\right) $$

We found that the test model was not significantly better than the null model (p = 0.61). Therefore, rather than collecting more data to satisfy our original criterion, we proceeded with the analyses over the full dataset collected thus far. Some information can be lost when dichotomizing a continuous variable like the self-motion measure we used for this cut-off criterion. Therefore, we further explored whether this measure was related to performance in any of the conditions. In these analyses, which are reported in detail in Appendix D, no evidence was found for such relationship. The code used for these analyses can be found on GitHub at: https://github.com/b-jorges/Motion-Perception-during-Self-Motion/blob/master/Final%20Paper%20Analysis%20Selfmotion.R.

#### Main hypotheses: Visually simulated observer motion

To test our main hypotheses regarding the influence of visually simulated observer motion on precision and accuracy, we performed the following tests over the main conditions, that is, when the wall backdrop was textured and the participant was moved visually, not the wall backdrop (“Simulated Observer Motion with Textured Backdrop” vs. “Static with textured backdrop”).

To assess the *Just Noticeable Difference (JND)* as a measure of precision, we first established a Test Model, in which responses were fitted to a cumulative Gaussian, with subject ID (“Subject”) and horizontal speed (Speed_horizontal_, with values -8, -6.6, 6.6, and 8 m/s) as random effects with random intercepts and random slopes for the speed of the ball cloud (“*Speed*_*Ball Cloud*_”) and the self-motion profile (“*Motion Profile*"), and self-motion (binary variable “*Subject Motion*” with the values “Yes” and “No”) and the speed of the ball cloud (“*Speed*_*Ball Cloud*_”) and their interaction as fixed effects. In lme4 syntax, this corresponds to:

4 $$ Response\sim Subject\ Motion\ast Spee{d}_{Ball\ Cloud}+\left( Spee{d}_{Ball\ Cloud}+ Motion\ Profile\right| Subject\Big)+\left( Spee{d}_{Ball\ Cloud}+ Motion\ Profile|\ {Speed}_{Target}\right) $$

We then established a Null Model with subject and horizontal speed as random effects with random intercepts, and subject motion profile and difference in speed between target and ball cloud as fixed effects, but not their interaction:

5 $$ Response\sim Subject\ Motion+ Spee{d}_{Ball\ Cloud}+\left( Spee{d}_{Ball\ Cloud}+ Motion\ Profile\right| Subject\Big)+\left( Spee{d}_{Ball\ Cloud}+ Motion\ Profile|\ {Speed}_{Target}\right) $$

We then used an ANOVA to test whether the test model was significantly better than the null model. If the interaction term improved the model significantly, the subject motion profile had a relevant influence on the slope of the fitted cumulative Gaussian. We expected the interaction parameter to be lower for Subject Motion = “Same Direction” and Subject Motion = “Opposite Direction,” thus putting into evidence that visually simulated observer motion decreases precision in object speed judgments during self-motion.

To assess the *Point of Subjective Equivalence (PSE)*, our Test Model contained the same random effects as above and the self-motion profile (ternary variable “*Motion Profile*” with the values “Same Direction,” “No Motion,” and “Opposite Direction”) and the speed of the ball cloud (“*Speed*_*Ball Cloud*_”) as fixed effects (Moscatelli et al., 2012). The lme4 syntax is:

6 $$ Response\sim Motion\ Profile+ Spee{d}_{Ball\ Cloud}+\left( Spee{d}_{Ball\ Cloud}+ Motion\ Profile\right|\  Subject\Big)+\left( Spee{d}_{Ball\ Cloud}+ Motion\ Profile|\ {Speed}_{Target}\right) $$

The Null Model contained the same random effects, and only the speed of the ball cloud as a fixed effect.

7 $$ Response\sim Spee{d}_{Ball\ Cloud}+\left( Spee{d}_{Ball\ Cloud}+ Motion\ Profile\right|\  Subject\Big)+\left( Spee{d}_{Ball\ Cloud}+ Motion\ Profile|\ {Speed}_{Target}\right) $$

We compared both models with an ANOVA and expected the Test Model to be significantly better than the Null Model, indicating that visually simulated observer motion had an impact on the PSE. Visually simulated observer motion in the same direction as the target should decrease perceived target speed and visually evoked self-motion in the opposite direction of the target should increase perceived target speed.

#### Control conditions

For the control conditions (*Textured Moving Backdrop* and *Blank Backdrop*), we used the model comparison [4]/[5] to assess whether wall motion (*Textured Moving Backdrop*) or visually simulated observer motion (*Blank Backdrop*), respectively, led to any biases in perceived velocity. For *Textured Moving Backdrop*, we expected that same pattern as for the main condition, but a slightly less complete compensation, with the untextured wall backdrop giving fewer cues about visually simulated observer motion. Furthermore, there should be next to no induced motion effects, which should augment the observed effect further. For *Blank Backdrop*, we expected a small effect of induced motion in the opposite direction of the effect of visually simulated observer motion, that is, an overestimation of speed when observer and wall moved in opposite directions, and an underestimation of speed when observer and wall moved in the same direction.

#### Power analysis

Based on the analysis plan above, we proceeded to a power analysis via simulation. We computed the power for the main condition (visually simulated observer motion with a textured wall backdrop). The R code used for this power analysis is available online on GitHub at: https://github.com/b-jorges/Motion-Perception-during-Self-Motion/blob/master/PowerAnalysisMotionEstimation.R. We first created datasets that would roughly resemble the data we are expecting to collect. At the core of the simulation of these datasets is the assumption that responses could be described by a cumulative Gaussian function (which approximates what is commonly known as “Psychometric Function”). The mean of the cumulative Gaussian corresponds to the PSE, and its standard deviation is proportional to the JND. We varied the means of the Gaussian according to the self-motion profile. Pilot data show consistently a bias to interpret the dot cloud as faster; when the observer is static, we thus assume a PSE of 2/3 of the presented speed. When the observer moved opposite to the target, we expected the PSE to be higher than in the static condition, and when the observer moved with the target, we expected the PSE to be lower. We conducted the power analysis assuming a difference of 1/8 of the mean presented speed of the visually evoked self-motion; Dokka et al. (2015) found biases up to 50% of the visually simulated observer motion. Their task, directionality judgments about downward motion with a lateral left- or rightward component, bears some similarities to ours, but is different enough to warrant a more conservative estimate for the sake of the power analysis. We also use a more naturalistic environment, which may render flow parsing more complete. For the standard deviation, we parted from a Weber fraction of 7% for the static condition (McKee, 1981), which corresponds roughly to a standard deviation of 10% of the PSE. Where the observer is moving, we expected increased JNDs and therefore an increased standard deviation. For the sake of this power analysis, we assume that the standard deviation in this case might be 1/4 higher than the standard deviation for a static observer. Dokka et al. (2015) found increases of up to 200% in thresholds from no self-motion to visually simulated observer motion. We choose a much more conservative value to account for task differences. Additionally, we varied the PSE and SD per participant by multiplying them with random values drawn from a normal distribution with a mean of 1 and a standard deviation of 0.1. To account for the fact that our staircase leads to a concentration of responses around the PSE, we drew the stimulus strengths from a Cauchy distribution with a location of 1 and a scale of 0.04. We drew 55 stimulus strengths for this distribution (per combination of target speed and self-motion, we use two PESTs with about 27 trials each; see above) and fed them into the cumulative Gaussian we established per condition and participant. This yielded the answer probability per trial. We then used these probabilities to draw binary answers (PEST faster yes/no) from a Bernoulli distribution for each trial.

We simulated 500 of these data sets, and conducted the analyses described above over each for 20, 22, 24, 26, 28, and 30 participants. We report the percentage where the Test Model was significantly better than the Null Model in Table 1.

**Table 1:** Simulated power values for 20, 22, 24, 26, 28, and 30 participants

N	Power accuracy	Power precision
*20*	1	0.744
*22*	1	0.800
*24*	1	0.812
*26*	1	0.852
*28*	1	0.876
*30*	1	0.910

While the effect should be easily detectable for the accuracy-based hypothesis, the precision hypothesis is somewhat harder to detect and requires at least 30 participants (for a power above 0.9). Note that, as the simulation process involves several sources of uncertainty, some variability is to be expected in the results, which explains why the power difference between 22 and 24 participants is smaller than the difference between 24 and 26 participants.

Our predictions for the effect of induced motion, which we want to probe for in the control conditions, are about accuracy. Considering that it is generally easier to detect accuracy differences than precision differences with the above method, we are confident that the participant number that allows us to detect the precision main effect will also allow us to detect any relevant effect of induced motion.

#### Pre-existing data

We collected data from seven pilot participants in the main condition (visually simulated observer motion with a texture wall backdrop). One (s07) was excluded because some of her PESTs did not converge. Two participants (s01 and s02) had previously done the task in 2D, but only their 3D data were included in the analysis. Pilot results are largely in line with our predictions: In terms of JNDs, we found that our Test Model was significantly better than the Null Model (p = 0.02), and effects trended in the direction of our hypothesis (regression coefficients of -0.078, SE = 0.034, for the interaction between visually simulated observer motion present and the difference in speed, which corresponds to a lower precision). For the PSEs, we found that our Test Model was significantly better than the Null Model (p < 0.001), and the effects were largely in the expected direction (regression coefficients of 0.072, SE = 0.05, for the main effect of congruent motion, and -0.25, SE = 0.053, for the main effect of incongruent motion; which corresponds to a lower perceived speed for congruent motion and visually evoked self-motion, and a higher perceived speed for incongruent motion and visually evoked self-motion). The code used for this analysis as well as the pilot data are available on GitHub (https://github.com/b-jorges/Motion-Perception-during-Self-Motion/blob/master/AnalysisPilotData.R).

The pilot data were not included in the final analysis; we recruited 30 new participants.

## Results

### Effect of visual self-motion on accuracy and precision – main experimental condition

The code for the following analyses can be found here (on GitHub: https://github.com/b-jorges/Motion-Perception-during-Self-Motion/blob/master/Final%20Paper%20Analysis.R).

#### Pre-registered analyses

**Fig. 3:**
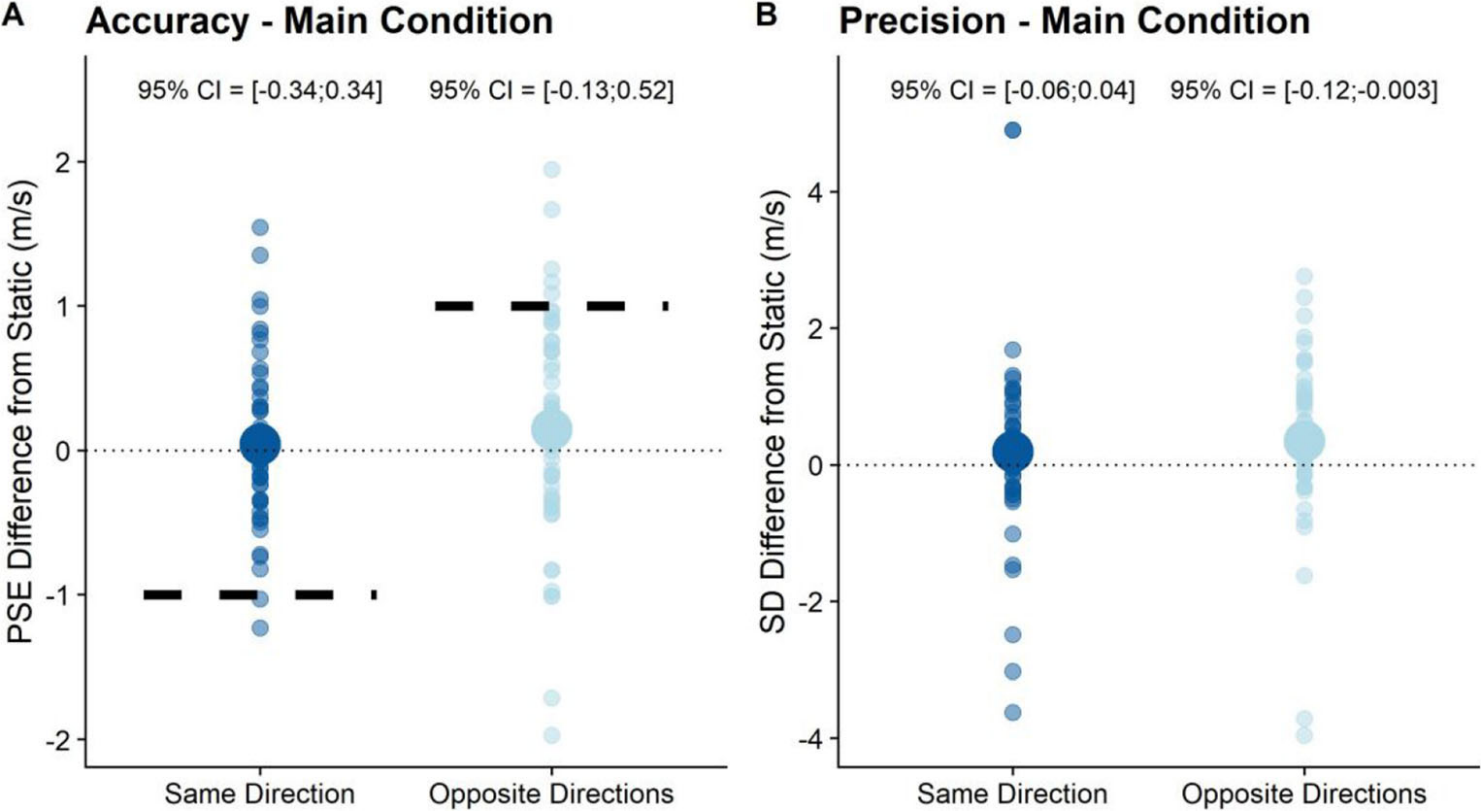
For illustration purposes, we fitted psychometric functions to the full staircase data for each participant, self-motion condition, motion profile, and *Speed*_*Target*_. We used the quickpsy package (Linares & López-Moliner, 2016) for R, which fits cumulative Gaussian functions by a direct likelihood minimization and yields the means of these functions as measure of accuracy and the standard deviations as a measure of precision. Please note that this was done only for data visualization as our statistical analysis sidesteps the need to fit individual psychometric functions for each staircase. **a** and **b** The big solid dots correspond to the difference between the mean PSEs (**a**) and standard deviations of the fitted psychometric functions (**b**) across participants and velocities in the “Same Direction” and “Opposite Directions” conditions and the “Static” condition, while the translucent dots illustrate individual PSEs or standard deviations per participant and *Speed*_*Target*_. **a** The dotted line also corresponds to 100% compensation for the Same Direction and Opposite Directions conditions. The dashed lines on the other hand correspond to the expected value for 0% compensation for self-motion, that is, when 100% of the visually simulated self-motion is added on or subtracted from the object motion

For our main hypotheses about the effects of visually simulated self-motion on precision and accuracy, we fitted the GLMMs specified above and used model comparisons to assess whether the variable of interest had a significant impact on performance (see Fig. 3). We first compared the precision test GLMM (Eq. 4) to the precision null GLMM (Eq. 5). A Likelihood Ratio Test (implemented in the anova() function from base R) showed that the test GLMM was not significantly better than the Null GLMM (p = 0.067), that is, we found no evidence that self-motion changed precision in the main experimental condition.

We then proceeded to testing the influence of the self-motion profile on PSEs by comparing the Test GLMM specified in Equation 6 to the Null GLMM specified in Equation 7. A Likelihood Ratio Test showed that the test GLMM was not significantly better than the null GLMM (p = 0.361), that is, our data failed to provide evidence that the motion profile had a significant impact on PSEs.

#### Exploratory analyses

We further conducted an exploratory analysis to assess whether precision was affected differentially by visually simulated self-motion in the same direction as the target and in the opposite direction of the target. To avoid having to subset the data, we computed bootstrapped 95% confidence intervals for all fitted regression coefficients rather than using model comparisons. We used the same GLMM as for the main confirmatory hypothesis, but rather than the variable “Self-motion: Yes/No” we used the variable “Motion Profile: Static/Same Direction/Opposite Directions” as the independent variable. We then used the confint() function from base R to compute the 95% confidence intervals for all fixed effects. See Table 2 for all regression coefficients, the respective standard errors, as well as the 95% confidence intervals. As evident from the table, only the regression coefficient pertaining to the influence of self-motion on precision in the Motion profile: Opposite Directions” was significantly different from zero (the interactions between the fixed effect “Speed_Ball Cloud” and “Motion Profile: Opposite Directions”). It was negative, that is precision in this this motion profile was lower.

**Table 2:** Regression coefficients, standard errors, and 95% confidence intervals for the Generalized Linear Mixed Model we set up to test for a differential impact of self-motion in the same direction as object motion and self-motion in the opposite direction of object motion

	Regression Coefficient	Standard Error	95% CI (lower)	95% CI (upper)	Significant
Intercept	-2.76	0.25	-3.26	-2.22	*
Self-motion: Same direction	0.03	0.16	**-0.34**	**0.34**	n.s.
Self-motion: Opposite directions	0.2	0.15	**-0.13**	**0.52**	n.s.
Speed (Ball Cloud)	0.59	0.05	**0.5**	**0.67**	*
Speed (Ball Cloud) * Self-motion: Same direction (Interaction)	-0.01	0.02	**-0.06**	**0.04**	n.s.
Speed (Ball Cloud) * Self-motion: Opposite directions (Interaction)	-0.06	0.03	**-0.12**	**-0.003**	*

In terms of PSEs, to explore a differential effect of visually simulated self-motion in the same direction as object motion and self-motion in the opposite direction as object motion, we used the 95% confidence intervals we computed for the Test GLMM fitted for precision. As evident from the regression coefficients and confidence intervals reported in Table 2, neither visually simulated self-motion in the same direction as the object nor in the opposite direction had a significant impact on PSEs (fixed effect “Self-motion: Same direction” and “Self-motion: Opposite direction,” respectively).

The regression coefficient can be interpreted as the mean degree of compensation for visually induced self-motion across all participants. A regression coefficient of -1 for “Same direction” and a regression coefficient of 1 for “Opposite direction” would mean no compensation for visually simulated self-motion, while regression coefficients of 0 would be synonymous with full compensation. Values beyond these limits would mean negative compensation or overcompensation, respectively. For the “Same Direction” motion profile, we thus found a compensation of 103%, that is, a very slight overcompensation, while we found an 80% compensation for the “Opposite Directions” motion profile.

### Effect of visual self-motion on accuracy and precision – control conditions

#### Blank Wall Condition: preregistered analyses

We first tested whether visually simulated self-motion had any impact on precision in the Blank Wall condition by fitting the Test GLMM (Eq. 4) and the Null GLMM (Eq. 5) to the relevant subset of the full dataset. A Likelihood Ratio Test showed that the Test GLMM was not significantly better than the Null GLMM (p = 0.379), indicating that visually simulated self-motion did not have a significant impact on precision in this condition.

We then tested whether visually simulated self-motion affected PSEs even with a blank wall backdrop. A Likelihood Ratio Test revealed that the Test GLMM (see Eq. 6) was not significantly better than the Null GLMM (see Eq. 7; p = 0.379), that is, we found no evidence that visually simulated self-motion influenced PSEs.

#### Blank Wall Condition: exploratory analyses

**Fig. 4:**
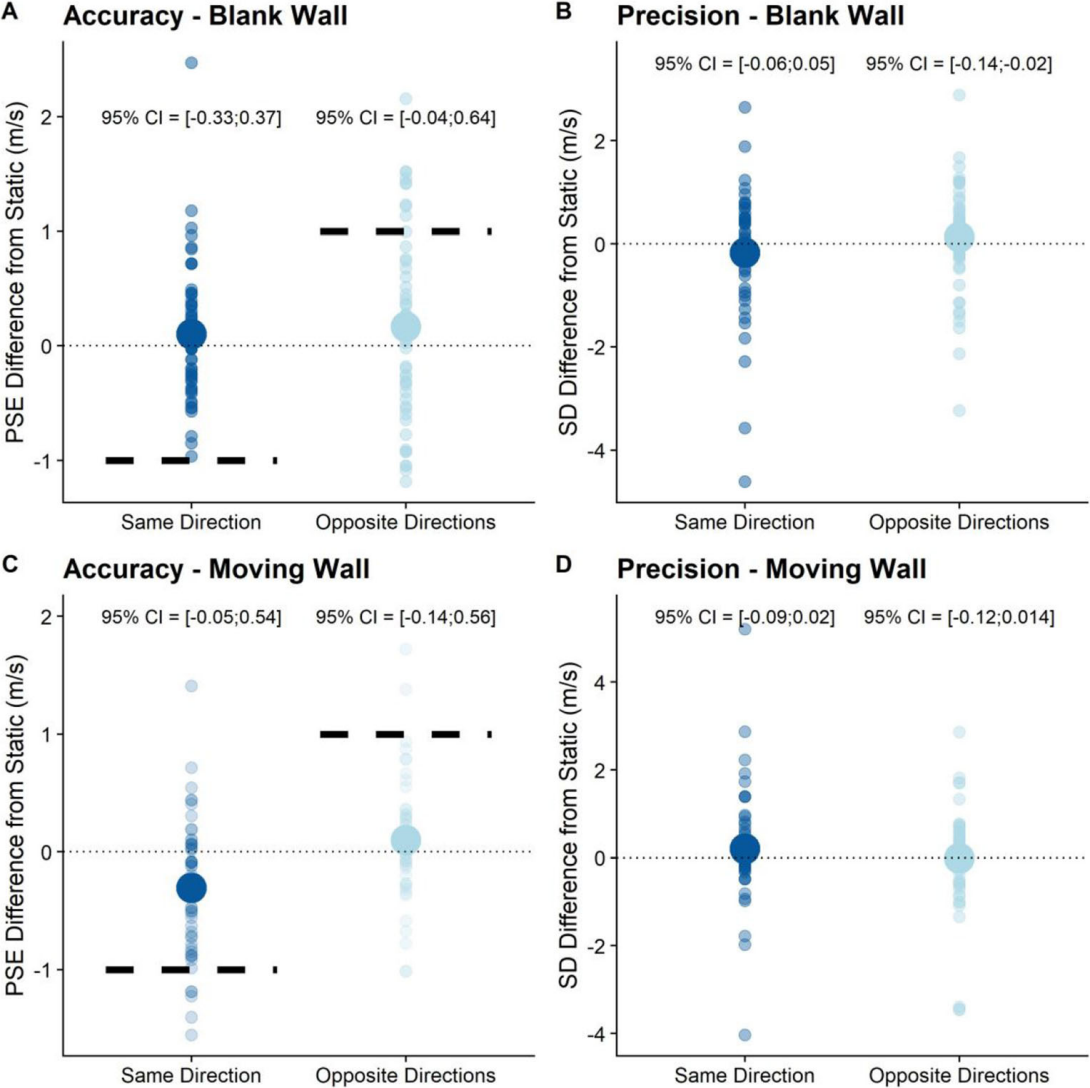
As for Fig. 3, but for the Blank Wall condition (**a** and **b**) and for the Moving Wall condition (**c** and **d**)

As for our main hypothesis, we explored to what extent visually simulated self-motion affected precision and accuracy differentially when simulated self-motion and object motion were in the same direction or in opposite directions (see Fig. 4a and b). As above, we computed 95% confidence intervals for all fixed effects in the full GLMM (specified in Eq. 4). The results for the blank wall subset of the dataset can be found in Table 3. Here, we found that visually simulated self-motion in the opposite direction of the target elicited significantly lower precision.

**Table 3:** Intercept and regression coefficients along with the corresponding standard errors and confidence intervals for the Generalized Linear Mixed Model we fitted to test for the impact of visually simulated self-motion with a blank wall backdrop on accuracy and precision

	Regression Coefficient	Standard Error	95% CI (lower)	95% CI (upper)	Significant
Intercept	-2.77	0.26	-3.33	-2.17	*
Self-motion: Same direction	0.06	0.15	**-0.33**	**0.37**	n.s.
Self-motion: Opposite directions	0.32	0.15	**-0.04**	**0.64**	n.s.
Speed (Ball Cloud)	0.59	0.05	**0.48**	**0.69**	*
Speed (Ball Cloud) * Self-motion: Same direction (Interaction)	-0.01	0.03	**-0.06**	**0.05**	n.s.
Speed (Ball Cloud) * Self-motion: Opposite directions (Interaction)	-0.08	0.02	**-0.14**	**-0.02**	*

As for compensation, visually induced self-motion in the same direction as the target was compensated for, on average, by 106%, that is, we observed a slight overcompensation. Visually induced self-motion in the opposite direction was compensated for by 68%.

#### Moving Wall Condition: preregistered analyses

Lastly, we tested whether the moving wall condition impacted precision and accuracy. To test for the impact of the moving backdrop on precision, we fitted the relevant GLMMs (Eqs. 4 and 5) and compared them with a Likelihood Ratio Test. In agreement with our hypothesis, the Test GLMM was not significantly better than the Null GLMM (p = 0.086), that is, the moving wall did not have a significant impact on precision.

For accuracy, we fitted the Test GLMM (Eq. 6) and the Null GLMM (Eq. 7) on the relevant subset of the data and compared them with a Likelihood Ratio Test. The Test GLMM was significantly better than the Null GLMM (p = 0.03), counter to our hypothesis that the moving wall would not impact PSEs. The intercept for the Test GLMM was -2.67 (SE = 0.26). We found regression coefficients of 0.09 (SE = 0.06) for the fixed effect “Self-motion: Same Direction,” -0.06 (SE = 0.08) for the fixed effect “Self-motion: Opposite directions,” and 0.57 (SE = 0.05) for “*Speed*_*Ball Cloud*_”.

#### Moving Wall Condition: exploratory analyses

Lastly, we again performed an exploratory analysis on whether wall backdrop motion in the same direction as the object affected accuracy or precision differently than wall backdrop motion in the opposite direction of the object (see Fig. 4c and d). As for the main hypothesis and the first control condition, we computed 95% confidence intervals for the GLMM specified as per Equation 4. The detailed results can be found in Table 4; no significant relationship was observed between any self-motion profile and accuracy or precision in this condition.

**Table 4:** Intercept and regression coefficients along with the corresponding standard errors and confidence intervals for the Generalized Linear Mixed Model we fitted to test for the impact of the moving wall backdrop on accuracy and precision

	Regression Coefficient	Standard Error	95% CI (lower)	95% CI (upper)	Significant
Intercept	-2.81	0.26	-3.37	-2.28	*
Self-motion: Same direction	0.24	0.13	**-0.05**	**0.54**	n.s.
Self-motion: Opposite directions	0.19	0.15	**-0.14**	**0.56**	n.s.
Speed (Ball Cloud)	0.6	0.05	**0.49**	**0.71**	*
Speed (Ball Cloud) * Self-motion: Same direction (Interaction)	-0.03	0.02	**-0.09**	**0.02**	n.s.
Speed (Ball Cloud) * Self-motion: Opposite directions (Interaction)	-0.05	0.02	**-0.12**	**0.01**	n.s.

## Modelling

Since we used a relatively ecological task, there are a number of different effects at play at the same time. Accuracy may be influenced not only by visually simulated self-motion but also by induced motion, while precision may be related to retinal velocities (which vary between the self-motion profiles) as well as the visually simulated self-motion. Therefore, we decided to prepare a model to allow to disambiguate the relative distributions of each of these effects and isolate the effects of self-motion. The four effects mentioned above, each impact performance differentially:

- Induced motion should lead to an overestimation of object speed when the object moves in the same direction as the observer, and to an underestimation of object speed when the object moves in the opposite direction. It should be present in the main experimental condition and in the moving wall condition, but it should be minimized in the blank wall condition.
- Self-motion should lead to an underestimation of object speed when the object moves in the same direction as the observer, and to an underestimation of object speed when the object moves in the opposite direction. It should be present in the main experimental condition and in the blank wall condition, but minimized in the blank wall condition.
- The retinal speed corresponding to object motion is higher when object and observer move in opposite directions than when the observer is static, and it is lower when object and observer move in the same direction. By Weber’s Law, the highest retinal velocities should elicit the lowest precision (in absolute terms) and vice versa.
- Self-motion requires the observer to parse out retinal stimulation due to self-motion to obtain an accurate representation of object speed. This process has been shown to add noise to the object speed estimate (Dokka et al., 2015). This effect should be present whenever the observer is moved visually through their environment.

We therefore built two models, one for accuracy and one for precision, to disentangle these effects. The implementation in R is available for download on GitHub at: https://github.com/b-jorges/Motion-Perception-during-Self-Motion/blob/master/Final%20Paper%20Modelling.R.

### Accuracy model

#### The Model

We used the following model:

8 $$ {PSE}_{Static}+{Effect}_{Self motion}\ast Conditio{n}_{Self- Motion}+\kern0.5em {Effect}_{Induced\ Motion}\ast {Condition}_{Induced\ Motion} $$

*PSE*_*Static*_ is the PSE in the static condition; we used the PSE in the static condition with the texture wall as baseline for the main experimental condition (textured wall, visually simulated self-motion) and the moving wall condition (texture wall, wall moves), and the PSE in the static Blank Wall condition (blank wall, visually simulated self-motion). *Effect*_*Selfmotion*_ captures how strongly visually simulated self-motion biased perception and is fitted to the data. *Wall Condition*_*Self* − *Motion*_ took a value of 1 when self-motion was expected to lead to an overestimation of speed (when self-motion and object motion were simulated in opposite directions), a value of -1 when an underestimation of speed was expected (when self-motion and object motion were simulated in the same direction) or a value of 0 when self-motion was not expected to bias speed perception (when no self-motion was simulated, including those conditions where the wall moved). *Effect*_*Induced Motion*_ is an indicator for the strength of the motion that our stimulus induced and is the second parameter that is fitted to the data. Lastly, *Condition*_*Induced Motion*_ could take the value 1 when the motion induced by the stimulus should augment perceived speed (i.e., either when the wall moved in the opposite direction to the stimulus in the Moving Wall Condition, or when the observer experienced visual self-motion in the same direction as the stimulus in the Main Experimental Condition); it took the value -1 when the motion induced by the wall backdrop should decrease perceived speed, such as when the wall moved in the same direction as the stimulus (in the Moving Wall Condition) or when visual self-motion in the opposite direction of the target motion was stimulated (in the Main Experimental Condition); and it took the value 0 when we expected no effect of induced motion, that is, when both wall and observer were still (in the Moving Wall and Main Experimental Condition, respectively) or when the wall backdrop was blank (in the Blank Wall Condition).

#### Fitting procedure

Fitting this model required the PSEs for each participant in each of the experimental conditions. For this reason, we excluded all participants for whom we had excluded one or more staircases in the initial outlier analysis. Twenty-one participants remained. We then proceeded to fitting the model by minimizing the root mean squared error (RMSE) between the model predictions and the observed PSEs for all conditions in each participant individually. We used the optim() function from base R (R Core Team, 2017) for the optimization with 0 as initial values for both *Effect*_*Selfmotion*_ and *Effect*_*Induced Motion*_. We thus obtained predictions for each staircase from each participant as well as values for *Effect*_*Selfmotion*_ and *Effect*_*Induced Motion*_ for each participant.

#### Results

**Fig. 5:**
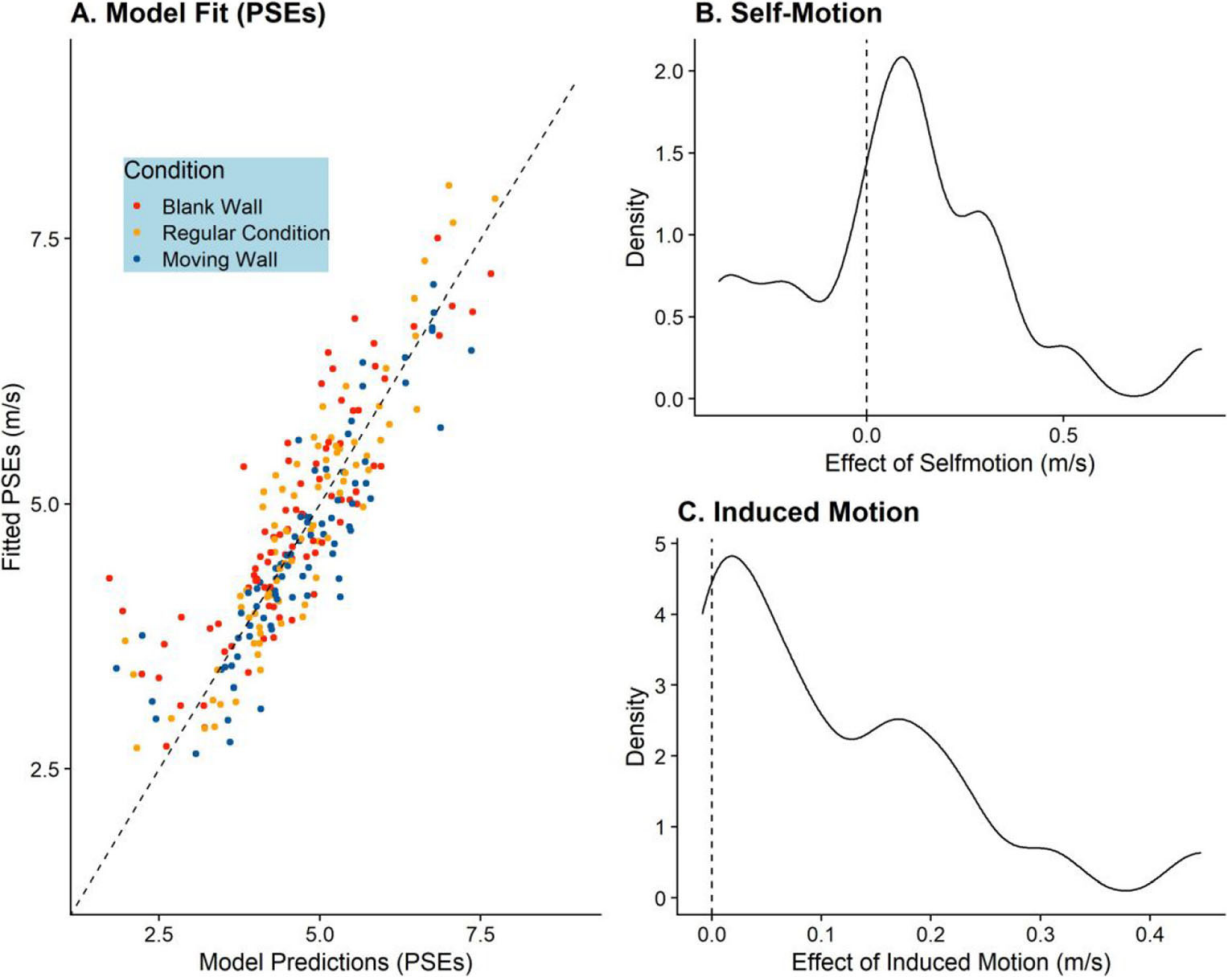
**a** Model predictions for the Point of Subjective Equivalence (PSE) model plotted against the PSEs observed in the participants. The dashed lines represent unity, where all the dots should fall if the model predicted performance perfectly. The different colors indicate the different experimental conditions (Main Experimental Condition, Blank Wall Condition, and Moving Wall Condition). **b** The distribution of *Effect*_*Selfmotion*_ for the 21 participants used to fit the model. **c** The distribution of *Effect*_*Induced Motion*_ for all participants included in this modelling exercise

As evident from Fig. 5a, our model makes qualitatively good predictions of the participants’ PSEs, and we obtained a mean RMSE of 0.37 m/s and a median RMSE of 0.44 m/s. For *Effect*_*Selfmotion*_, we found a mean value of 0.09 m/s and a median value of 0.11 m/s. That is, across the whole population participants compensated for about 90% of the self-motion (which we had simulated at 1 m/s). However, as evident from Fig. 5b, there was a large spread of individual differences. For *Effect*_*Induced Motion*_, we found a mean value of 0.11 m/s and a median value of 0.09 m/s, that is, 1 m/s wall movement elicited about 0.1 m/s of motion. The distribution of this value can be found in Fig. 5c.

### Precision model

#### The model

We constructed the model as follows:

9 $$ {SD}_{Static}+{Effect}_{Self- Motion}\ast {Condition}_{Self- Motion}+{Effect}_{Retinal\ Velocity}\ast Retinal\ Velocity $$

where *SD*_*Static*_ is the standard deviation of the psychometric function in the Static conditions, again, as above, separately for the Blank Wall Condition and the Main Experimental Condition. *Effect*_*Self* − *Motion*_ is a free parameter that captures the extent to which visually simulated self-motion lowers or raises precision (as measured by the standard deviation of the fitted psychometric functions). This parameter is fitted to the data. *Condition*_*Self* − *Motion*_ can take the value 1, when visual self-motion was simulated (same direction and opposite direction trials both in the Blank Wall Condition and the Main Experimental Condition), or 0 when no self-motion was simulated (including Moving Wall Condition). *Effect*_*Retinal Velocity*_ is also fitted to the data and captures how the retinal speed of the stimuli predicts differences in standard deviations of the fitted psychometric functions. *Retinal Velocity* is the retinal speed over the 0.5 s a trial lasted. See Table 5 for the exact values for each condition.

**Table 5:** Mean retinal speeds throughout the 0.5 s of presentation of the big target in the different motion profiles and conditions

Condition	Target speed	Retinal Speed
Self-motion simulated in same direction as target	6.6 m/s	27.6 °/s
	8 m/s	32.5 °/s
Self-motion simulated in opposite direction as target	6.6 m/s	73.3 °/s
	8 m/s	82.2 °/s
No self-motion simulated (including Moving Wall Condition)	6.6 m/s	46.6 °/s
	8 m/s	56.2 °/s

#### Fitting procedure

We used the same procedure as for the PSE model, with the only difference being that we used the standard deviations of the fitted psychometric functions (as a proxy for precision) as target instead of the measured PSEs.

#### Results

**Fig. 6:**
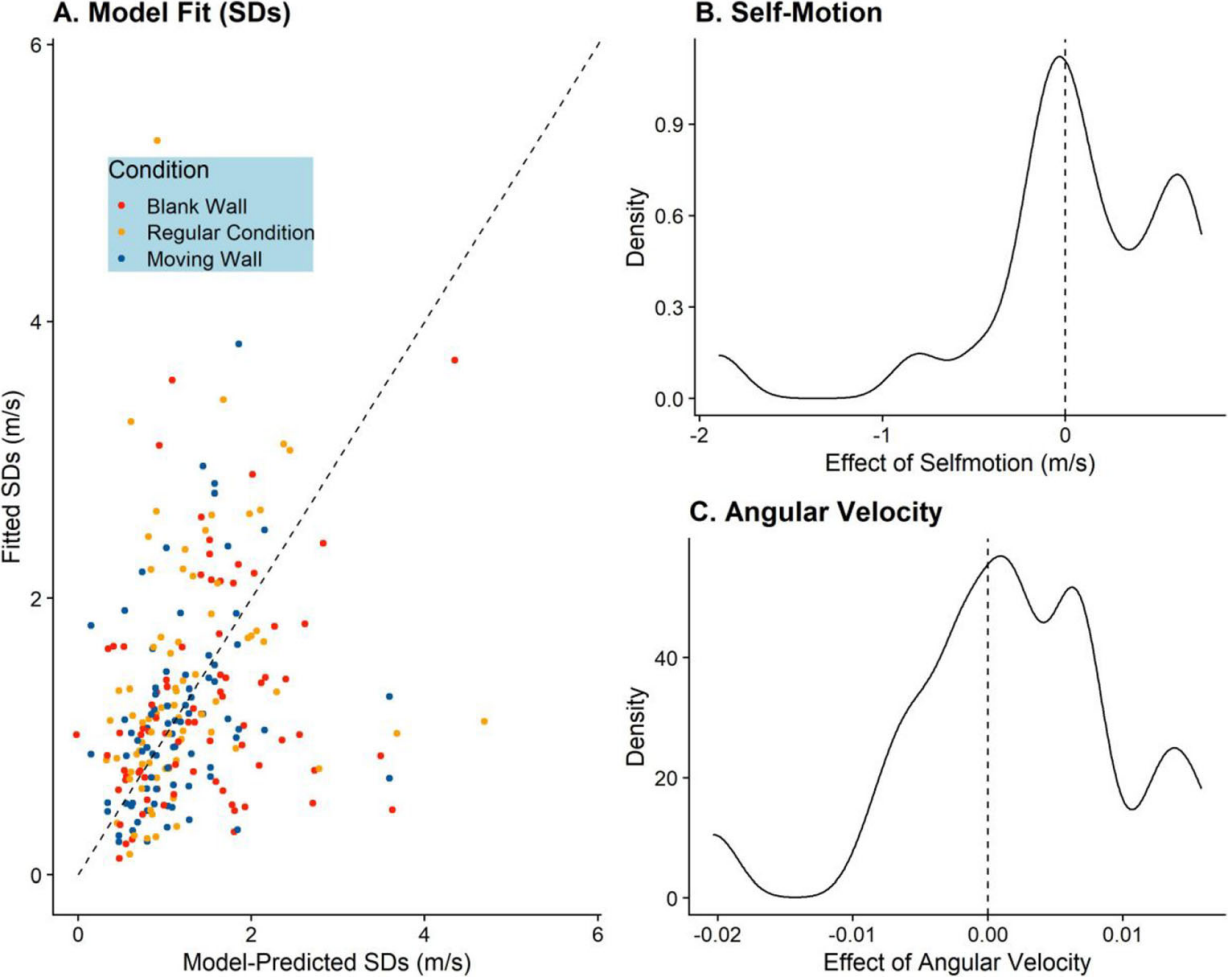
**a** As for Fig. 5a, but for the predicted and observed standard deviations of the fitted psychometric functions. **b** Distribution of fitted values for *Effect*_*Self* − *Motion*, *JNDs*_. **c** Distribution of fitted values for *Effect*_*Retinal Velocity*_

Qualitatively, the fit of this model was worse than for the PSE model. The mean RMSE across all participants is 0.75 m/s and the median RMSE is 0.6 m/s. Please see Fig. 6a for an illustration of the fit. We found a mean *Effect*_*Self* − *Motion*_ of 0.031 m/s, the median being 0.025 m/s. That is, the standard deviations of the fitted psychometric functions were higher by a very small amount in response to visual self-motion. SD For *Effect*_*Retinal Velocity*_**,** the mean was 0.0016 °/s, and the median was 0.0017 °/s. Higher retinal velocities corresponding to the target were thus connected with slightly lower precision overall, albeit the spread across participants was large. Note also that the retinal velocities were two to three orders of magnitude higher than any effect we observed here. Please refer to Figs. 6b and 6c for the full distributions of fitted values.

Lastly, to test whether the relationship between compensation for self-motion and precision found by Dokka and her colleagues (2015) held in our dataset as well, we tested the correlation between *Effect*_*Self* − *Motion*, *PSEs*_ and *Effect*_*Self* − *Motion*, *JNDs*_. We found a non-significant regression coefficient of -0.2, indicating that higher compensation for visually induced self-motion was (descriptively) related to lower precision, even though this correlation was not significantly different from zero (p = 0.187). However, keep in mind that we only obtained one value per participant, and we used a reduced dataset to fit this model, that is, we have an effective sample size of 21. A failure to detect a correlation may thus be due to lacking power rather than the absence of a relationship.

## Discussion

We did not find evidence that participants’ perception of object speed was biased by visually simulated self-motion. That is, they generally compensated effectively for visual self-motion. We found evidence for an (on average) full compensation both in the Main Experimental Condition (103%) and the Blank Wall Condition (106%) when self-motion was simulated visually in the same direction as the target motion. For visual self-motion in the opposite direction of the target, we found a descriptively lower degree of compensation: 80% in the Main Experimental Condition and 68% in the Blank Wall Condition. Please note that these were, however, not significantly different from a complete (100%) compensation, with 95% confidence intervals of [48; 112] for the main experimental Condition and [36; 104] in the Blank Wall Condition. While we did find a significant effect of induced motion on PSEs in the Moving Wall Condition, we could not pinpoint in which motion profile this effect occurred in subsequent exploratory analyses. It has been suggested that motion is only induced when the inducing stimulus and the target are in the same plane of fixation (Heckmann & Howard, 1991); since in our case the inducing stimulus (the wall) was behind the target, albeit at a relatively small distance (the wall was simulated 2 m behind the target, which in turn was simulated 8 m in front of the observer), this may have weakened the effect of induced motion in our experiment. Fitting the PSE model showed similarly that participants compensated to a large extent for visually simulated self-motion, albeit with a large between-participant variability, while induced motion seemed to influence performance as well, equally subject to a large between-participant variability. This high variability may in part be related to the fact that our participants completed the experiment in their own home without direct supervision by the researchers. While this should, in principle, not bias results, it may well increase the variability in responses, both within and between participants.

Further, we found that precision was significantly lower for visually simulated self-motion in the opposite direction of object motion, both in the Blank Wall Condition and in the Main Experimental Condition. The Moving Wall Condition, on the other hand, did not elicit a decrease in precision. This indicates that precision was indeed impacted by visually simulated self-motion rather than because of the relative movement of the backdrop and the stimulus. Since the retinal speed corresponding to the target was lower when self-motion was simulated visually in the same direction as the target than when the observer was static, and it was higher for visual self-motion in the opposite direction of the target, according to Weber’s Law, precision (in absolute terms) should be higher in the former and lower in the latter case. To help disentangle the effect of Weber’s Law and the effect of self-motion on precision, we fitted another model to the precision data. This model indicated, surprisingly, that the effect of Weber’s Law played only a very small role in our experiment: that is, we did not find evidence that lower retinal speeds were connected to meaningful changes in precision. Similarly, the effect of visually simulated self-motion on precision was found to be extremely small on average and highly variable from participant to participant.

Our findings in term of compensation for visually simulated self-motion are to some extent in line with the findings of Dokka et al. (2015) for a different task meant to assess the same perceptual process: in their experiment, a target was presented moving vertically with only a small horizontal component. During presentation, participants could experience lateral self-motion visually, vestibularly, or both visually and vestibularly at once, and had to judge on each trial whether the horizontal velocity component of the target was directed towards the left or towards the right. The authors found a lack of compensation for self-motion in all conditions: vestibular cues alone did not allow participants to compensate for self-motion, visual cues led to a compensation of 47% and combined visual and vestibular cues enabled a compensation of 58%. For our purely visual self-motion, we found a more complete compensation: when observers were moved visually in the same direction as the target, compensation was on average complete, while visual self-motion in the opposite direction of the target was compensated at 80% when the wall backdrop in the environment was textured (and led thus presumably to a more accurate self-motion estimate), and 68% when the wall backdrop was blank. Dokka et al. (2015) found also that a more complete compensation for self-motion was related to lower precision. Apart from the differences in the task, one notable difference between our study and Dokka et al. (2015) was that they projected their visual stimulus onto a wall in front of the participant and the environment was a starfield. We, on the other hand, immersed participants in a 3D environment that was rich in visual cues to self-motion. It thus stands to reason that our more realistic, immersive environment allowed participants to flow parse more successfully and recover a more accurate representation of target speed even during visually simulated self-motion. An alternative reason for why our participants compensated more fully for visual self-motion could be the Gaussian self-motion profile we simulated. In principle, if participants solved the task based on the retinal speeds they observed in the very beginning and very end, where the simulated self-motion speed was very low, while ignoring the faster part in the middle, they could solve this task with a reasonable degree of accuracy without taking into account self-motion at all. However, this would have to occur within the first and/or last 50 ms, when the visually simulated self-motion speed was negligible. Human speed discrimination is much less precise when motion is presented for less than 100 ms (McKee & Welch, 1985). It thus seems unlikely that our participants used such strategy. We further were not able to replicate the negative correlation between completeness of compensation and precision observed by Dokka et al. (2015). While we did find a trend in the same direction, this trend was not statistically significant, which may, however, be due to the relatively low power in our statistical assessment of this relationship.

Some other studies have assessed completeness of compensation for self-motion and precision changes in response to self-motion in a variety of tasks. Niehorster and Li (2017) found incomplete compensation for visual self-motion in a retinal motion nulling paradigm similar to the one employed by Dokka et al. (2015). While this lack of compensation was found consistently for a variety of displays that manipulated the availability of local and global motion cues, none of these displays aimed to provide an ecological environment for the observer. Further, Xie et al. (2020) used the same paradigm to assess the contributions of visual and non-visual cues to flow parsing. They found that visual cues alone evoked compensation for about 80% of the self-motion, with non-visual cues alone accounting for 50%, and practically full compensation was observed when both types of cues were combined. A similar observation was made by Dyde and Harris (2008), who also found incomplete compensation when self-motion was presented only visually. Both studies, however, used displays that were less ecological than ours, which may explain why, under some circumstances, our participants were able to compensate fully for self-motion even though self-motion was presented only visually. Finally, Probst et al. (1986) observed higher thresholds (that is, lower precision) in the detection of object-motion for different types of self-motion induced by vestibular, visual, or cervico-somatosensory stimulation, again with a rudimentary display providing self-motion cues. It is thus likely that the completeness of compensation for self-motion is affected by the richness of visual cues to self-motion, with more ecological displays such as ours facilitating a more accurate self-motion estimate and thus more complete compensation. Similarly, self-motion might lead to lower precision particularly when self-motion cues are available only in one modality (e.g., visually) and/or the visual environment is impoverished.

Lastly, did our participants interpret the visually simulated self-motion as we expected them to? Participants either perceived themselves as moving and the world as static or the world as moving and themselves as static; and they could judge object motion relative to the world or relative to themselves. The logical space for this question consisted thus of the combination of each of these possibilities, that is: (1) world static and motion judged relative to world (intended); (2) world moving and motion judged relative to the world; (3) world static and motion judged relative to observer; (4) world moving and motion judged relative to observer. The latter two interpretations would predict shifts of the PSE by exactly 1 m/s, that is, we would expect PSEs to be concentrated around the dashed lines in Figs. 3a and 4a. While a few participants did display this behavior, visual inspection suggests that the majority compensated at least to some extent for visual self-motion. That is, we found little evidence that participants judged motion relative to themselves. For scenario (2), we would expect no shifts in PSEs between the “Static” motion profile and the “Same Direction” and “Opposite Directions” profiles. And, indeed, we found no significant differences between these conditions. Furthermore, when assessing participants’ sense of self-motion in the environment explicitly, we found that some of them perceived the world as moving rather than themselves as moving (see Fig. 2). However, the majority of the participants did interpret the visual stimulation as self-motion, even though only about half of them satisfied our strict criterion of judgments above 0.6 on a scale of -1 (only world motion perceived) to 1 (only self-motion perceived). Additionally, when testing the hypothesis that participants who satisfied this criterion performed differently in our main task from participants who did not, we found no evidence for a difference in performance between both groups. Nonetheless, the possibility remains that we were not able to capture significant biases in response to visual self-motion because participants interpreted the world as moving and judged object motion relative to the world.

## Conclusions

We set out to study to what extent visually simulated self-motion might bias perceived lateral object motion and lead to lower precision. We found no biases at all when visual self-motion and target motion went in the same direction, while visual self-motion was compensated for by about 80% when they went in opposite directions. Precision was slightly lower when self-motion was simulated visually in the opposite direction to the target, but we found no evidence for a decrease when observer and target moved in the same direction. While we have discussed some caveats, such as the possibility that participants misinterpreted self-motion as world motion, we attribute our participants’ ability to compensate for the retinal motion introduced by our simulation of their self-motion as being largely due to the rich, ecologically valid nature of our virtual reality display.

## Appendix

### List of Deviations from Preregistration

In the following, we disclose all changes with regards to the preregistration of this study.

A series of changes have been made to allow for data collection during the COVID-19 pandemic of 2020-2021. We packaged the experiment such that individuals who owned VR equipment would be able to run the experiment by themselves from the safety of their homes. To this end, we modified the original experiment as follows:

- To reach a larger group of potential participants, we adjusted our code to allow for testing on all VR devices capable of running Unity executables, rather than limiting it to Oculus Rift CV1 as originally planned.
- Instead of a finger mouse, responses were given with the keyboard.
- We prepared detailed instructions (in PDF and video format) that allowed participants to administer the experiment to themselves at home.
- Rather than undergrads, we tested participants who were recruited by word of mouth, on online communities, and through the company XpertVR.
- Instead of the Stereo Fly stereo vision test, we programmed a custom stereo-blindness test that we presented in VR. In this program, we showed participants 20 trials that each consisted of two rectangles. They were asked to judge which of the two were closer. One of the rectangles was presented slightly closer to the observer and we adjusted the size of this rectangle such that its retinal size was the same. With that, participants could only rely on disparity, which we kept constant at 200 arcseconds across all 20 trials, to solve the task. More information on the reasoning behind this task, as well as the underlying math, and on how to download the Unity project and the executable can be found on GitHub at: https://github.com/b-jorges/Stereotest.

We further implemented the following changes that were unrelated to remote testing:

- We failed to include an outlier analysis in the original preregistration. We remedied this oversight by concluding a very liberal outlier analysis, and we also included the results we obtain without excluding any data in the appendix.
- We added some exploratory analyses that are clearly marked as such.
- We added a modelling section to ascertain quantitatively, rather than just qualitatively, to what extent the effects of visually simulated self-motion, induced motion, and retinal velocities (by virtue of Weber’s Law) impacted results.
- We noticed that the results of our statistical analysis did not match the plotted data (either in strength or in direction of the effect). We re-ran the analyses and substituted some of the independent variables in the model specifications; we switched the difference in speed between the test stimulus and the comparison stimulus for the speed of the comparison stimulus both as fixed effects and as random effects. We also added random slopes for motion profile per participant and target speed. Using Likelihood Ratio Tests, we confirmed that the model fits were better for this configuration, which is an indicator of less biased results. We report both the model comparisons and the results of the preregistered analyses in Appendix B.

### Deviations from original analysis plan

When comparing the results of the planned analysis with the plotted data, we noticed certain incompatibilities. Misspecifications of fixed and random effects in Generalized Linear Mixed Models are known to bias results. In the analyses reported in the body of the paper we therefore used a different dependent variable (the speed of the ball cloud rather than the difference in speed between the ball cloud and the target ball) and a more complete set of random effects (the speed of the ball cloud and the self-motion profile rather than the difference in speed between the ball cloud and the target ball). To determine the adequate model specification, we compared four different setups, the preregistered one (see Wilkinson & Rogers’ notation in Eq. 10 below); one where we used the velocity of the ball cloud instead of the difference in speed between ball cloud and target ball (see Eq. 11 below); one where we added random slopes for the Motion Profile per Participant and per Speed of the target ball (see Eq. 12 below); and one where we made both changes (see Eq. 13 below).

10 $$ Response\sim Motion\ Profile\ast Difference+\left( Difference\right| Subject\Big)+\left( Difference|\ {Speed}_{Target}\right) $$

11 $$ Response\sim Motion\ Profile\ast Spee{d}_{Ball\ Cloud}+\left( Spee{d}_{Ball\ Cloud}\right| Subject\Big)+\left( Spee{d}_{Ball\ Cloud}|\ {Speed}_{Target}\right) $$

12 $$ Response\sim Motion\ Profile\ast Difference+\left( Difference+ Motion\ Profile\right| Subject\Big)+\left( Difference+ Motion\ Profile|\ {Speed}_{Target}\right) $$

13 $$ Response\sim Subject\ Motion\ast Spee{d}_{Ball\ Cloud}+\left( Spee{d}_{Ball\ Cloud}+ Motion\ Profile\right| Subject\Big)+\left( Spee{d}_{Ball\ Cloud}+ Motion\ Profile|\ {Speed}_{Target}\right) $$

To assess that these model specifications were a better fit than the ones proposed in the original preregistration, we used Likelihood Ratio Tests, which allow us to compare the fit of two statistical models and ascertain which is more adequate for the data at hand.

We fitted these models for each condition (Main Experimental Condition, Blank Wall Condition, Moving Wall Condition) separately and compared successively whether Model 11 was significantly better than Model 10, then if Model 12 was better than Model 11, and finally if Model 13 was better than Model 12. We found that Model 11 was better than Model 10 for all conditions (with p < 0.001 for all conditions). Model 12 was better than Model 11 for the Main Experimental Condition (p = 0.036) and the Blank Wall Condition (p = 0.002), but not for the Moving Wall Condition (p = 1). For the Moving Wall Condition, we therefore compared Model 13 to Model 11. For the other two conditions, we compared Model 13 and Model 12. For the Main Experimental Condition and the Blank Wall Condition, Model 13 was better than Model 12 (p < 0.001), and for the Moving Wall Condition, Model 13 was better than Model 11 (p = 0.039). We thus used the model specification laid out in Equation 13 for all analyses.

### Main results according to the original analysis plan

For full transparency, we further report the results when following the original analysis plan, that is, using the model specification described in Equation 10 and without any outlier analysis.

*Main experimental condition*

When following the original data analysis plan (without removing any outliers and using the difference between test and comparison speed as independent variable), the test model for precision (Eq. 4) was still significantly better than the null model (Eq. 5; p < 0.001). In the same vein, the PSE test model (Eq. 6) was significantly better than the PSE null model (Eq. 7; p = 0.006). See Table 6 for regression coefficients, standard errors and 95% confidence intervals for the corresponding exploratory analyses for the full model (Eq. 4) used for the exploratory analyses.

**Table 6:** As for Table 2, but for the full dataset without removing any outliers

	Regression Coefficient	Standard Error	95% CI (lower)	95% CI (upper)	Significant
Intercept	1.53	0.2	1.23	1.91	*
Self-motion: Same direction	-0.07	0.06	**-0.18**	**0.07**	n.s.
Self-motion: Opposite directions	-0.35	0.06	**-0.48**	**-0.22**	*
Difference	0.58	0.04	**0.51**	**0.66**	*
Difference * Self-motion: Same direction (Interaction)	-0.02	0.02	**-0.07**	**0.03**	n.s.
Difference * Self-motion: Opposite directions (Interaction)	-0.11	0.02	**-0.15**	**-0.06**	*

*Blank Wall Condition*

The precision test model (Eq. 4) was again better than the corresponding null model (Eq. 5; p = 0.028) for the Blank Wall Condition, and the PSE test model (Eq. 6) was better than the PSE null model (Eq. 7; p = 0.022) as well. Table 7 displays the regression coefficients, standard errors, and 95% confidence intervals for the full model (Eq. 4) used for the exploratory analyses.

**Table 7:** As for Table 3, but for the full dataset without removing any outliers

	Regression Coefficient	Standard Error	95% CI (lower)	95% CI (upper)	Significant
Intercept	1.41	0.18	1.07	1.79	*
Self-motion: Same direction	0.03	0.06	**-0.09**	**0.16**	n.s.
Self-motion: Opposite directions	-0.22	0.05	**-0.33**	**-0.1**	*
Difference	0.55	0.04	**0.47**	**0.63**	*
Difference * Self-motion: Same direction (Interaction)	0	0.02	**-0.04**	**0.06**	n.s.
Difference * Self-motion: Opposite directions (Interaction)	-0.07	0.02	**-0.11**	**-0.02**	*

*Moving Wall Condition*

The precision test model (Eq. 4) was significantly better than the null model (Eq. 5; p < 0.001) for the Moving Wall Condition and the PSE test model (Eq. 6) was significantly better than the null model (Eq. 7; p < 0.001). In Table 8, we detail the regression coefficients, standard errors, and 95% confidence intervals for the full model (Eq. 4) used for the exploratory analyses.

**Table 8:** As for Table 4, but for the full dataset without removing any outliers

	Regression Coefficient	Standard Error	95% CI (lower)	95% CI (upper)	Significant
Intercept	1.49	0.19	1.17	1.81	*
Self-motion: Same direction	0.02	0.06	**-0.12**	**0.16**	n.s.
Self-motion: Opposite directions	-0.19	0.06	**-0.31**	**-0.07**	*
Difference	0.58	0.04	**0.5**	**0.67**	*
Difference * Self-motion: Same direction (Interaction)	-0.03	0.02	**-0.08**	**0.02**	n.s.
Difference * Self-motion: Opposite directions (Interaction)	-0.06	0.02	**-0.1**	**-0.01**	*

*Summary*

When comparing the results as per the initial analysis plan, without outlier analysis and with the original GLMM setup (as presented in Appendix C), to the results presented in the main paper, stark differences can be observed. As shown in Appendix B, our initial analysis plan, particularly with regards to the model specifications of the Generalized Linear Mixed Models, was inadequate and produced biased results. The correct model specifications performed much better, with better model fits across the board. We therefore believe that the results reported in this Appendix C should be discarded completely.

### The link between the continuous measure of self-motion perception and performance

In addition to the analysis we performed to assess the impact of the self-motion inclusion criterion (see “Outlier Analysis and Exclusion Criteria”), we further explored whether there might be a relationship between the continuous self-motion ratings and performance in terms of PSEs and JNDs. To avoid triple interactions, we fitted GLMMs of the following structure to each of the motion profiles (“Same direction” and “Opposite Directions”) and wall conditions (“Regular Wall”, “Blank Wall” and “Moving Wall”) separately:

14 $$ Response\sim Spee{d}_{Ball\ Cloud}\ast Self- motion\ Score+\left( Spee{d}_{Ball\ Cloud}\right|\  Subject\Big)+\left( Spee{d}_{Ball\ Cloud}|\ {Speed}_{Target}\right) $$

We compared each of these models to the corresponding null model without the self-motion score as factor:

15 $$ Response\sim Spee{d}_{Ball\ Cloud}+\left( Spee{d}_{Ball\ Cloud}\right|\  Subject\Big)+\left( Spee{d}_{Ball\ Cloud}|\ {Speed}_{Target}\right) $$

We then compared each test model (as per Eq. 14) to the corresponding null model (as per Eq. 15) with a Likelihood Ratio Test. We found that the test model was not significantly better than the null model for any of the conditions, with p values of 0.62 for Same Direction in the Regular Wall condition, 0.97 for Opposite Direction in the Regular wall condition, 0.83 for Same Direction in the Blank Wall condition, 0.9 for Opposite Direction in the Blank Wall condition, 0.47 for Same Direction in the Moving Wall condition and 0.4 for Opposite Direction in the Moving Wall condition. This indicates that our data provided no evidence that the self-motion score was significantly related to performance.
